# Retrospective Clinical Evaluation of a Decision-Support Software for Adaptive Radiotherapy of Head and Neck Cancer Patients

**DOI:** 10.3389/fonc.2022.777793

**Published:** 2022-06-30

**Authors:** Sebastien A. A. Gros, Anand P. Santhanam, Alec M. Block, Bahman Emami, Brian H. Lee, Cara Joyce

**Affiliations:** ^1^ Loyola University Chicago, Loyola University Medical Center, Stritch School of Medicine, Department of Radiation Oncology, Cardinal Bernardin Cancer Center, Maywood, IL, United States; ^2^ Department of Radiation Oncology, University of California, Los Angeles, Los Angeles, CA, United States; ^3^ Department of Public Health, Stritch School of Medicine, Loyola University Chicago, Maywood, IL, United States

**Keywords:** adaptive radiotherapy, head and neck cancer, clinical workflow, deformable image registration (DIR), prediction model

## Abstract

**Purpose:**

This study aimed to evaluate the clinical need for an automated decision-support software platform for adaptive radiation therapy (ART) of head and neck cancer (HNC) patients.

**Methods:**

We tested RTapp (SegAna), a new ART software platform for deciding when a treatment replan is needed, to investigate a set of 27 HNC patients’ data retrospectively. For each fraction, the software estimated key components of ART such as daily dose distribution and cumulative doses received by targets and organs at risk (OARs) from daily 3D imaging in real-time. RTapp also included a prediction algorithm that analyzed dosimetric parameter (DP) trends against user-specified thresholds to proactively trigger adaptive re-planning up to four fractions ahead. The DPs evaluated for ART were based on treatment planning dose constraints. Warning (V_95_<95%) and adaptation (V_95_<93%) thresholds were set for PTVs, while OAR adaptation dosimetric endpoints of +10% (DE_10_) were set for all D_max_ and D_mean_ DPs. Any threshold violation at end of treatment (EOT) triggered a review of the DP trends to determine the threshold-crossing fraction ***Fx*** when the violations occurred. The prediction model accuracy was determined as the difference between calculated and predicted DP values with 95% confidence intervals (CI_95_).

**Results:**

RTapp was able to address the needs of treatment adaptation. Specifically, we identified 18/27 studies (67%) for violating PTV coverage or parotid D_mean_ at EOT. Twelve PTVs had V_95_<95% (mean coverage decrease of −6.8 ± 2.9%) including six flagged for adaptation at median ***Fx*** = 6 (range, 1–16). Seventeen parotids were flagged for exceeding D_mean_ dose constraints with a median increase of +2.60 Gy (range, 0.99–6.31 Gy) at EOT, including nine with DP>DE_10_. The differences between predicted and calculated PTV V_95_ and parotid D_mean_ was up to 7.6% (mean ± CI_95_, −2.7 ± 4.1%) and 5 Gy (mean ± CI_95_, 0.3 ± 1.6 Gy), respectively. The most accurate predictions were obtained closest to the threshold-crossing fraction. For parotids, the results showed that ***Fx*** ranged between fractions 1 and 23, with a lack of specific trend demonstrating that the need for treatment adaptation may be verified for every fraction.

**Conclusion:**

Integrated in an ART clinical workflow, RTapp aids in predicting whether specific treatment would require adaptation up to four fractions ahead of time.

## 1 Introduction

The use of intensity-modulated radiation therapy (IMRT) and volumetric-modulated arc therapy (VMAT) techniques to treat head and neck cancer (HNC) enables the delivery of highly conformal radiotherapy (RT) treatments with complex dose distributions. Initial issues in RT delivery, such as patient setup and localization errors, were addressed by the International Commission on Radiation Units and Measurements (ICRU) in the 1980s and 1990s with the recommendations for the delineation of gross tumor volume (GTV), clinical target volume (CTV), and planning target volume (PTV) structures (1–3) and minimized by the continuous improvement of imaging modalities for patient setup verification. The latest image-guided radiation therapy (IGRT) solutions have pushed the limits of reducing PTV-to-CTV margins to only a few millimeters (4–6), generating a greater sparing of organs at risk (OARs). However, these smaller margins leave very little room for errors, as an inadequate PTV coverage could lead to treatment failure. The proximity of critical structures to GTVs, the change in volume, the displacement of targets and OARs, and weight loss during treatment are now the new challenges faced by radiation oncologists, as they all constitute risks for target under-dosage, leading to possible local failure or radiation toxicities (7–11). Successful strategies to improve HNC patients’ quality of life after RT include sparing the parotid glands (PGs) and the mandible to decrease the risks of xerostomia (12) and osteoradionecrosis (13).

Adaptive radiotherapy (ART) involves all methods that aim to adapt RT treatments and delivered dose distributions to any specific patient anatomical changes (14). The main potential benefits of ART are to ensure the adequate dosimetric coverage of targets and to limit OAR doses throughout the treatment, assuming that it will increase the therapeutic ratio and provide better outcomes for cancer patients (15). ART encompasses offline, online, and real-time strategies to mitigate the effects of anatomic shifts and setup errors (16). While both offline and online ART involve imaging to review the current anatomy and to assess the need for a new plan, most common ART methods allowed by current technologies for HNC patients are variants of offline strategies (17–22), as these are well suited for the slow progressing nature of anatomic changes observed during HNC RT treatments (8, 20, 23). Online methods are more appropriate to address the effects of stochastic patient setup errors (24, 25), such as those caused by the inter-fractional variation in shoulder position (26, 27) or the loose fitting of the immobilization mask due to weight loss. They are, however, only commercially available for HNC treatment on dedicated adaptive RT systems such as the Halcyon with Ethos (28) (Varian, Palo Alto, CA), Radixact (29) (Accuray, Sunnyvale, USA), or the MRIdian (Viewray, Cleveland, USA) and Unity (Elekta, Stockholm, Sweden) combined MRI-linac platforms (30). MR-guided online ART is a promising avenue for HNC treatment, as it would allow for daily online adaptation (important for fast responding HN tumors) and may allow for the online monitoring of tumor response with functional MRI protocols without the additional dose delivered with nuclear imaging (30). Real-time ART introduces additional sophisticated patient monitoring to correct intra-fractional anatomic shifts in real time during treatment delivery (31) and seems excessive for HNC treatments. In contrast, offline ART can readily be implemented with the current basic clinical RT treatment resources.

Clinical offline ART workflows follow four key steps: imaging, assessment, re-planning, and quality assurance (QA) (16). It is recommended that HNC patients be monitored with CT or CBCT imaging acquired frequently (daily or weekly) throughout the treatment (32, 33). These images are reviewed by a radiation oncologist who then decides whether the treatment plan is to be adapted. The re-planning decision is based on a review strategy, which typically consists of assessing anatomic variations and their impact on the dosimetry of targets and OARs. After the registration of the periodic CBCT images to the initial plan CT, an initial qualitative evaluation visually compares the structure contours from the periodic CBCT images against pretreatment volumes. Subsequent quantitative assessments require specialized software tools to compute similarity and distance metrics between the initial volumes and the new structures and to estimate the treatment dose from the most current patient anatomy. The determination of patient- and plan-specific thresholds based on treatment site, fractionation, and outcome is key to optimize the ART workflow and to provide an individualized approach well suited for HNC patients. However, the ART tasks performed after periodic patient imaging requires several hours of expert physicians, physicists, and dosimetrists, consuming resources that most radiation oncology facilities cannot afford. As no commercial automated ART offline workflow is yet available with gantry-mounted linacs, quantitative changes for targets and OAR structures of interest cannot be estimated in a feasible time for the majority of HNC patients. The time-consuming nature of offline ART workflows leads to delays until the new plan is available for treatment. Consequently, clinicians might continue to administer RT according to the original treatment plan, which may reduce the efficacy of the radiation treatment sought by triggering a plan adaptation. Therefore, there is a current need for an automated and quantitative framework that will process the daily imaging, generate the contoured structures, compute the dose to be received by the structures of interest, and predict if a re-planning is required to maintain the current plan quality. Such automated workflow would ideally include the implementation of predictive models to allow for the instantiation of clinical adaptive re-planning ahead of time.

This manuscript reports on our experience with a newly developed commercial decision-support software platform for ART, RTapp™ (SegAna, Orlando, FL), which automatically tracks and analyzes daily anatomical changes throughout an entire course of RT and predicts when treatment plans will exceed dose constraints. Most software tasks are optimized to run on a graphics processing unit (GPU) and allow the presentation of the results in near real time (19). A feasibility study of the ART workflow introduced by RTapp was conducted retrospectively with HNC patient data to assess if RTapp could help determine the need for adaptive re-planning during treatment, based on a set of hypothetical PTV coverage and OAR dose thresholds.

## 2 Materials and Methods

### 2.1 Retrospective Cohort

#### 2.1.1 Enrollment Criteria

An initial set of 81 HNC patients treated with external beam radiation therapy (EBRT) between January and December 2019 with VMAT was surveyed for the retrospective analysis, under Institutional Review Board (IRB) protocol (# LU213253). Exclusion criteria included patients who received prior HNC RT (n=2) or EBRT with sequential boost (n=4) or treatment adaptation (n=4), as these require a new CT scan; patient not imaged with daily kV-CBCT (n=4); and any patient for whom complete PTV and PG volumes were not included in the CBCT field of view (n=24) for all treatment fractions due to the need to track dose constraints based on full volume coverage. Additional exclusion criteria were applied to sinus and nasal cavity sites (n=3) and lips (n=3) due to target locations with initial limited interest for adaptation. The above selection criteria were fulfilled by 37 patients, out of which 27 were randomly selected for analysis. The diversity of HNC sites (**Table 1**) was representative of the HNC patient population treated at our institution. **Table 2** summarizes the distribution of treatment prescriptions and targets included in this study.

**Table 1:** Distribution of HNC sites from the patient cohort.

Diagnoses per Sites	# Cases
Pharynx	17
Oral cavity	3
Larynx	5
Salivary glands	1
Lymph nodes	1

**Table 2:** Treatment plans prescriptions from patient cohort.

SIB Doses (Gy)	Dose per fraction (Gy)	Total # fractions	Targets	# cases
60/54	2/1.8	30	Post-operative bed/nodal basin	9
70/63/56	2/1.8/1.6	35	GTV/CTV/nodal basin	15
66/60/54	2.2/2/1.8	30	GTV/CTV/nodal basin	1
66/54	2.2/1.8	30	GTV/CTV	1
59.4/54.12	1.8/1.64	33	High risk mucosa & ipsilateral node basin/at risk contralateral nodes	1

All patients received their treatment in a simultaneous integrated boost (SIB) setting.

#### 2.1.2 Treatment Planning, Delivery, and Imaging

The treatment planning images were acquired on a 32-slice Siemens SOMATOM CT Open AS scanner (Siemens Healthineers, Erlangen, Germany) with a reconstructed slice thickness of 3 mm and metal artifact reduction (MAR) enabled by default. All HNC patients were immobilized with a Q-fix Fiberplast^®^ Portrait S-frame Head and Shoulder thermoplastic immobilization mask (Qfix, Avondale, PA). The GTV and nodal CTV targets were contoured by the treating physician prior to applying 2–3 mm PTV margins defined as follow: high-risk (HR) PTV, intermediate-risk (IR) PTV, and low-risk (LR) PTV. A dosimetrist contoured all normal structures and OARs. The plan optimization followed the list of dose constraints required by the treating physician for each individual plan. All patients were treated on a Varian Truebeam linear accelerator (Varian Medical Systems, Palo Alto, CA) with 6 MV VMAT in 30–35 fractions. Daily patient setup and verification was performed with the On-Board kV Imaging (OBI) system. Each CBCT image set was comprised of 93 frames with 2 mm separation. **Table 3** lists the planning dose constraints for our patient cohort. Each patient dataset, composed of treatment plan CT, structure set, 3D dose, and daily 3D kV-CBCTs, was anonymized prior to be exported as DICOM RT objects for processing by RTapp.

**Table 3:** Treatment planning dose constraints and structure specific dosimetric endpoints (DE) .

Organ/volume of interest	Parameter	Planning goal	Warning DE	Adaptation DE
PTVs	V_95_	>95%	95%	93%
	Hotspot	<110% Rx dose	110%	110%
Spinal cord	D_max_	<45 or 50 Gy	Planning goal	10%
Brainstem	D_max_	<45 or 50 Gy	Planning goal	10%
Oral cavity	D_max_	No hotspot	110%	110%
Spared parotid^a^	D_mean_	<20 Gy	Planning goal	10%
Contralateral parotid^b^	D_mean_	N/A	Planning goal	10%
Cervical esophagus	D_max_	No hotspot	110%	110%
Mandible	D_1cc_	65–75 Gy	Planning goal	Planning goal
Cochlea	D_mean_	<35 Gy	Planning goal	10%
Larynx	D_max_	No hotspot	110%	110%
Brachial plexus	D_max_	65 or 66 Gy	Planning goal	Planning goal

The DEs serve as thresholds to trigger the plan review for adaption in an adaptive radiotherapy workflow with RTapp.

a If planning goal achieved, DE relative to 20 Gy. If not and D_mean_ < 21 Gy, DE are relative to 21 Gy.

b If planned PG D_mean_ < 26 Gy then DE relative to 26 Gy.

DE, dosimetric endpoint.

### 2.2 Overview of the Adaptive Software Platform

RTapp is a stand-alone and vendor agnostic application. As such, it can be employed with any treatment delivery platform, as long as 3D imaging is available for analysis.

#### 2.2.1 Software Front-End

**Figure 1:**
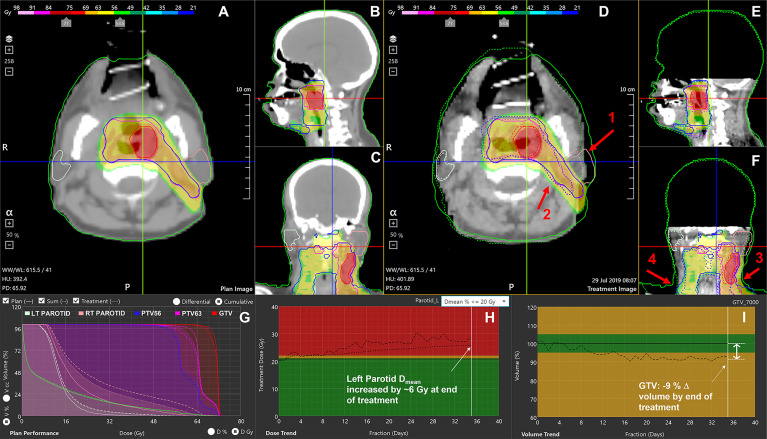
RTapp software user interface. **(A–C)** Initial plan CT, structures, and 3D dose. **(D–F)** The final fraction’s CBCT with plan structures (solid line), deformed contours to current day’s position (dotted line), and initial 3D dose. **(G)** DVH showing the plan dose (solid line), delivered dose up to the current fraction (DVH_sum_, dotted line), and day of treatment dose scaled to full treatment (DVH_day_, dashed line). **(H)** Trend for parotid’s mean dose; **(I)** tumor volume regression throughout the treatment. The red arrows indicate notable effects of RT and weight loss on the left parotid (1), PTVs (2), external neck contour (3), and inconsistent shoulders repositioning (4).

The front-end of the application (**Figure 1**) displays treatment-specific data, anatomic visualization windows (**Figures 1A–F**) and panels with graphical data (**Figures 1G–I**) to guide the ART decision-making process for the selected patient study.

#### 2.2.2 RTapp Workflow

**Figure 2:**
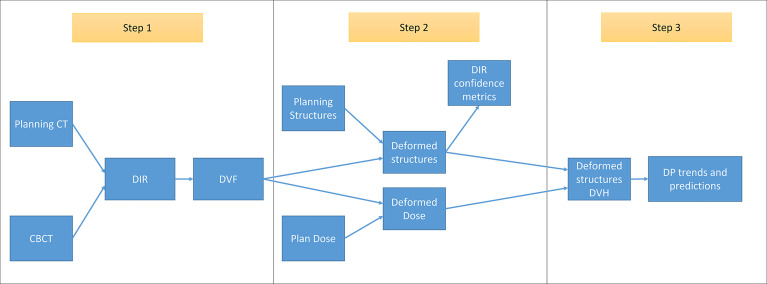
Schematic of RTapp workflow divided into three steps: 1, generation of the deformation vector field (DVF) from the deformable registration of planning CT to daily CBCT anatomy; 2, deformation of initial plan structures and dose based on daily DVF; and 3, generation of deformed structures DVH and trend and prediction of dosimetric parameters.

The main purpose of RTapp is to estimate the dose received by each structure at any treatment time point. A predictive algorithm analyzes the trend of user-defined structure and specific dosimetric parameters (DPs) against predetermined dosimetric endpoint (DE) values to forecast if, and so when, any dose constraint would be violated. The automated RTapp workflow can be divided in three steps, as outlined in **Figure 2**.

*Step 1*. For each treatment fraction, the initial treatment plan CT images and structures are deformed to match the daily setup CBCT images. An optical flow-based deformable image registration (DIR) algorithm (34) automatically registers the initial and daily 3D image sets and generates a deformation vector field (DVF).

*Step 2*. The DVF is then employed to deform the initial plan structures and dose into daily deformed structures and dose, as described in Qi et al. (19). The deformation results can be verified *via* DIR confidence metrics.

*Step 3*. The daily dose distribution within any structure is calculated from the deformed structures and dose. The dose volume histograms (DVHs) for the day of treatment (DVH_day_) and up to the current treatment time are generated from the estimated dose distribution (**Figure 1G**). The day of treatment DVH represents the daily dose scaled to the whole course of treatment, assuming that the daily anatomy would be maintained for the whole course of treatment. The sum DVH (DVH_sum_) summarizes structure doses accumulated up to the current fraction, scaling the latest fraction dose to the remaining course of treatment, assuming that the most current structure anatomy and dose distribution would hold for the remaining treatment fractions. Specific dosimetric parameters DP_day_ and DP_sum_ are calculated from the DVH_day_ and DVH_sum_ to populate dose trend graphs displayed on the software front-end (**Figure 1H**). A linear predictive model analyzes the trends of DP_sum_ to forecast their values over the next four fractions, hence providing quantitative data to guide the decision to adapt proactively.

#### 2.2.3 Implementation Environment

RTapp was tested as a standalone application installed on a Microsoft Windows 10 workstation equipped with a 2.3-GHz intel Core i-9 CPU, 32 GB RAM, and a Nvidia GeForce RTX2070 (8 GB RAM). The processing of a single fraction data set (93 CBCT images and ~30 structures) typically took <1 min.

### 2.3 Evaluation of DIR Quality

The quality of the deformation was first assessed by visually comparing overlaid initial and deformed structures contours on the CBCT viewing panels (**Figures 1D–F**). A quantitative evaluation was then performed with two DIR confidence metrics provided by RTapp, following the recommendations from the AAPM TG-132 report (35). Structures with normalized cross-correlation (NCC) values <0.85, or for which the displacement vector of the secondary image voxels of a structure exceeded a 7-mm “large” displacement threshold after deformation, were automatically flagged for review. While the NCC threshold was hard coded into RTapp, the pixel displacement threshold of 7 mm was selected, as it qualitatively provided the best trade-off between too many flags (<5 mm) and missing registration errors (>9 mm) due to positioning errors on an initial test HNC patient data set. The DIR algorithm parameters were adjusted before reprocessing a fraction when the flagged structures deformations were assessed as inaccurate after user review.

### 2.4 Dosimetric Metrics and Thresholds for Target Coverage and OARs

The structures monitored for the retrospective study were all PTV, CTV, GTV, and nodal targets, and parotid glands (PGs), spinal cord, cochleae, brainstem, mandible, esophagus, and larynx when applicable. The DPs evaluated against the need for adaptation were based on the dose constraints required for the treatment planning of HNC at our institution (**Table 3**). All cases had identical requirements for the PTVs (V_95_ > 95% of prescription dose; maximum dose D_max_ < 110%) and for the PGs (D_mean_ < 20 Gy). Additional PG DPs were defined specifically for this study: D_mean_ < 21 Gy for cases where the dosimetrist could not keep the mean ipsilateral PG dose below 20 Gy due to the overlap with IR PTVs, and D_mean_ < 26 Gy for spared contralateral PGs. Other OAR constraints varied per plan.

### 2.5 Adaptive Review Strategy

Each patient data set was fully processed with RTapp. The DVH_day_ and DVH_sum_ generated by RTapp for the final dose (**Figure 1G**) were compared to the initial plan DVH (DVH_plan_). Any violation of dose constraints at end of treatment (EOT) were tallied as potential case for adaptation. For every dose constraint violation, the DP_sum_ trend (**Figure 1H**) was reviewed to determine the fraction when the violation occurred. For this work, a hypothetical “warning” threshold (V_95_ < 95%) was chosen to investigate the impact of daily setup variation on PTV coverage, and an “adaptation” threshold of −2% (V_95_ < 93%) was set to trigger a review of this patient’s anatomy for replanning. A hypothetical OAR “adaptation” dosimetric endpoint of 10% (DE_10_) was set uniformly for all D_max_ and D_mean_ DPs. A set of HNC structure-specific endpoints, which lists the above structures and associated adaptation thresholds, was saved in RTapp to conduct this retrospective work.

### 2.6 Prediction Model Accuracy

The accuracy of the prediction model was evaluated by calculating the difference between the DP_sum_ value at the fraction when it violated the adaptation threshold and the predicted pDP_sum_ values from the four fractions preceding the violation time point. The difference in DP_sum_ was averaged over all patient studies flagged for adaptation to calculate the 95% confidence interval as summarized by Equation 1. For a particular DP_sum_ value crossing a threshold at fraction *Fx*, the difference in DP_sum_ from a predicted pDP_sum_ value based on processed fraction [*Fx* − *i*] with *i* = [4,3,2,1], averaged over all *n* flagged studies is given by:


(1) $${\left[\Delta {\text{DP}}_{\text{sum}}\right]}_{Fx-i}=\frac{1}{n}\sum_{n}\left({\left[{\text{pDP}}_{\text{sum}}\right]}_{Fx-i,n}-{\left[{\text{DP}}_{\text{sum}}\right]}_{Fx}\right)$$

Finally, this retrospective work was devised to help provide estimates on the proportion of HNC patients expected to need ART and gather information on the magnitude of differences observed between plan results and actual determined DP values in order to help better plan future prospective analysis with this software. As such, a potential clinical ART workflow integrating RTapp in the treatment of HNC patients was proposed based on our experience.

## 3 Results

### 3.1 Adaptive review

**Figure 3:**
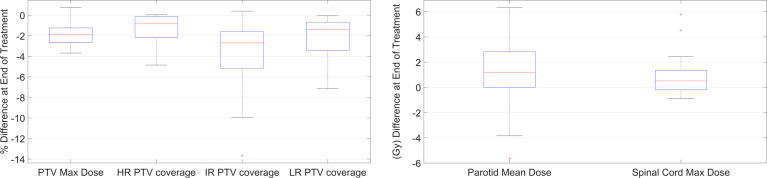
Boxplot summary of the DP values differences between start and end of treatment for PTVs, PGs, and spinal cord.

The retrospective analysis with RTapp reported 18/27 patient studies (67%) that failed to meet at least one dose constraint at EOT. The flagged structures were 12 PTV targets and 17 PGs. Other structure DPs remained below their warning threshold values throughout the treatments. Box plots summarizing the differences between the DVH_plan_ and the DVH_sum_ derived PTV D_max_ and V_95_, and PG D_mean_ and spinal cord D_max_ are shown in **Figure 3**. Overall, PTV dosimetry and coverage decreased while PG and spinal cord doses increased during RT, with the latter remaining below thresholds.

### 3.2 Targets Coverage

The difference in V_95_ between planned and EOT DP_sum_ values ranged from 0 to −4.9% for HR PTVs, +0.4 to −13.7% for IR PTVs, and −0.1 to −7.1% for LR PTVs. The difference in PTV D_max_ ranged from +0.74 to −3.7 Gy. While all PTVs met the minimum V_95_ > 95% coverage requirement after treatment planning, 12 PTVs belonging to nine patients were flagged for under-coverage (V_95_ < 95%) at EOT. **Table 4** summarizes the changes in targets coverage. The mean PTV coverage decrease was $\text{Δ}{\bar{\text{V}}}_{95}=-6.8\pm 2.9\;\%$ resulting in a mean final ${\bar{\text{V}}}_{95}=91.8\pm 2.9\%$ . Six flagged targets were IR PTVs, accounting for the initial GTVs, involved nodal basin, and areas of microscopic spread (studies 94, 118, 146, 46, 19, and 18). Four flagged PTVs were covering postoperative beds (studies 15, 8, and 99), while the last three flagged PTVs were covering low-risk nodal basin (studies 8, 18, and 19). The example shown in **Figure 1**, from study 118, illustrates the effect of internal anatomical changes and weight loss on the last fraction CBCT (**Figures 1D–F**). The inwards shift and the regression of the deformed PTV contour are responsible for the loss of coverage (dotted line DVH_sum_ on **Figure 1G**) for IR PTV63 with an EOT V_95_ value of 92.86%.

**Table 4:** PTV coverage for patient studies with V_95_ < 95% coverage at end of treatment.

Study #	Target	Initial V_95_	Final V_95_	Δ V_95_	Fraction for V_95_ < 95%	Cumulative dose (Gy)
94	IR PTV60 GTV+nodes	100.00%	93.74%	−6.26%	10	20
118	IR PTV63 GTV+nodes	99.43%	92.86%	−6.57%	16	32
146	IR PTV63 GTV+nodes	99.43%	93.55%	−5.88%	1	2
46	IR PTV63 GTV+nodes	99.27%	94.76%	−4.51%	35	70
15	PTV60	98.98%	90.03%	−8.95%	3	6
8	PTV60 Post−op bed	98.12%	84.45%	−13.67%	9	18
8	PTV54 Nodal basin	98.36%	92.17%	−6.19%	13	26
99	PTV60 Post−op bed	94.67%	93.27%	−1.40%	1	2
19	IR PTV63 GTV+nodes	99.38%	94.21%	−5.17%	31	62
19	LR PTV56 GTV+nodes	96.29%	89.15%	−7.14%	2	4
18	IR PTV63 GTV+nodes	99.50%	89.56%	−9.94%	1	2
18	LR PTV56 GTV+nodes	99.97%	93.81%	−6.16%	3	6

The fraction at which the coverage crossed the 95% warning threshold, and the corresponding cumulative dose were identified from the V_95_ trend.

### 3.3 Trend Analysis of Target Dosimetric Parameters

**Figure 4:**
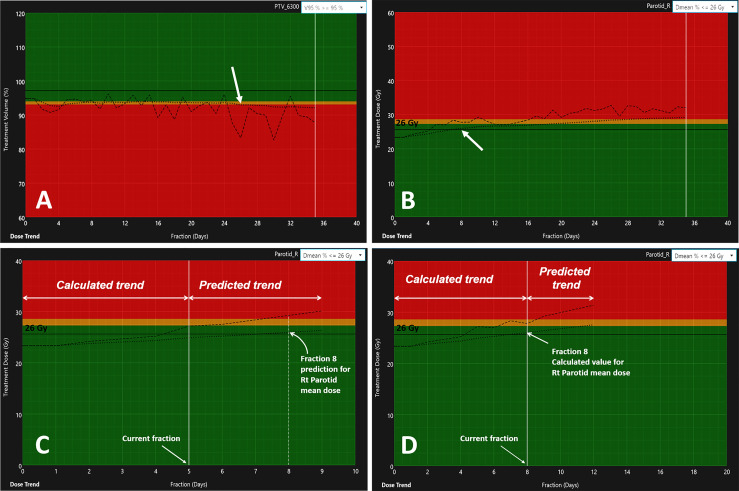
Variation of dosimetric parameters over the full treatments for **(A)** the V_95_ trend of PTV63 (study 118) and **(B)** the increase in PG D_mean_ for the right PG (study 81). The dashed lines indicate the DP_day_ value estimated from the day of treatment DVH_day_. The dotted line represents the variation of the DP_sum_ estimated from the cumulative DVH_sum_. The arrows indicate the fraction at which the DP_sum_ values reached their respective adaptation thresholds: V_95_ < 93% at fraction 26 for PTV63 **(A)** and PG D_mean_ > 26 Gy at fraction 8 **(B)**. Panels **(C, D)** illustrate the trend prediction in DP_sum_ for the right PG. The data to the left of the current fraction represent calculated values from daily deformed anatomy. The data to the right predict the variations of the DP_day_ and DP_sum_ for the next four fractions.

The threshold-crossing fraction for V_95_ < 95% threshold was extracted from the automated fraction processing reports (**Table 4**, “Fraction for V_95_ < 95%”). The results hinted at a clustering of PTV coverage constraint violations occurring either during the first three fractions (early) or after the second quarter of the treatment (late), with median final V_95_ values of 91.6% and 92.5%, respectively. However, an independent samples two-tailed t-test for equality of the mean final V_95_ values of both groups indicated no significant difference (p = 0.799). The offline review of daily setup CBCT images revealed that local setup errors were responsible for the lack of V_95_ coverage at the first fraction due to head and mandible tilts (studies 18 and 19) and shoulder misalignment (studies 15, 99, and 146). The fractions at which a specific PTV crossed the V_95_ < 93% “adaptation” threshold were determined from their respective V_95_ DP_sum_ trend graphs. **Figure 4A** shows an example dose trend from study 118 where the PTV coverage decreases during treatment and crossed the 93% “adaptation” threshold (indicated by the red background) at fraction 26, as pointed out by the arrow. Six PTVs from studies 8 (PTV60 and PTV54), 15 (PTV60), 18 (IR PTV63), 19 (LR PTV56), and 118 (IR PTV63) were flagged for adaptation before EOT with V_95_ < 93% at a median fraction of 6 (range, 1–16).

### 3.4 Parotid Mean Dose

The difference between the planned PG D_mean_ and the DP_sum_ value estimated at EOT by RTapp ranged from −5.6 to +6.3 Gy, with 74% of the parotids showing an increase in overall PG dose. RTapp reported 13 patients (17 flagged PGs) with at least one PG exceeding their dose constraint. Eleven studies had at least one PG crossing the 20 Gy D_mean_ threshold at EOT. Three cases with an initial PG D_mean_ between 20–21 Gy were reported for exceeding a 21-Gy D_mean_ by EOT. Three additional contralateral PGs for which the initial plan kept the PG D_mean_ < 26 Gy were also flagged. The median increase in PG mean dose at end of treatment was +2.60 Gy (range, 0.99–6.31 Gy) for all 17 flagged PGs. The mean difference between start and EOT for PGs violating the 20, 21, and 26 Gy D_mean_ constraint were +2.92, +3.49, and +2.51 Gy, respectively.

### 3.5 Trend Analysis of Parotid Dosimetric Parameters

**Table 5** summarizes all dose constraints (DE_0_) and DE_10_ violations for the PGs. The fraction at which the PGs mean doses exceeded their respective 10% deviation thresholds was obtained from the automated fraction processing reports (**Table 5**, “fraction for D_mean_ > DE”). The flagged studies can be divided into two independent groups. (1) Eight patient studies had a PG DP_day_ failure occurring within the first two treatment fractions, with an average PG mean dose difference ΔD_mean_ = 4.05 ± 1.46 Gy. These occurred too early to result from radiation treatment and were most likely due to patients relaxing in their immobilization mask, leading to inconsistent patient setup throughout the course of treatment. (2) The second group comprises nine studies with endpoint failures occurring later during treatment (median threshold-crossing fraction *Fx*=20; range, 8–30), with an average PG mean dose difference ΔD_mean_ = 1.98 ± 0.78 Gy, likely to result from gradual body weight loss and internal anatomical shifts induced by radiation treatment response. **Figure 4B** presents the D_mean_ trend from the right PG (study 81), indicating the 26 Gy DE_0_ being exceeded at *Fx* = 8. An independent samples two-tailed t-test for equality of the mean PG dose difference between the early and late groups showed significance (p = 0.005). Nine PGs were flagged for adaptation with PG D_mean_ > DE_10_ before EOT, with average PG mean dose differences ΔD_mean_ = 4.68 ± 1.11 Gy and ΔD_mean_ = 2.54 ± 0.78 Gy for the early (N=6) and late (N=3) groups, respectively.

**Table 5:** Parotid glands D_mean_ violations of the warning dose thresholds (DE_0_) and of the 10% dosimetric endpoints (DE_10_) for triggering adaptation.

Study no.	SIB doses (Gy)	Initial plan D_mean_ (Gy)	Final RTapp D_mean_ (Gy)	Δ D_mean_ (Gy)	Laterality	DE_0_ (Gy)	Fraction for D_mean_ > DE_0_	Cumulative dose for D_mean_ > DE_0_	Fraction for D_mean_ > DE_10_	Cumulative dose for D_mean_ > DE_10_
46	70/63/56	19.66	22.25	2.60	Left	20	2	4	22	44
81	70/63/56	19.54	25.85	6.31	Left	20	1	2	1	2
79	70/63/56	19.98	23.17	3.19	Left	20	1	2	1	2
137	70/63/56	19.63	24.00	4.37	Left	20	2	4	4	8
94	66/60/54	19.40	20.95	1.55	Right	20	12	24	20	40
105	66/54	19.24	20.23	0.99	Right	20	21	42	–	–
22	70/63/56	19.56	20.87	1.20	Right	20	30	60	–	–
26	70/63/56	19.91	20.11	2.29	Right	20	16	32	–	–
78	60/54	19.57	21.86	2.29	Left	20	9	18	–	–
116	60/54	17.62	20.29	2.68	Right	20	16	32	–	–
83	70/63/56	19.74	25.44	5.7	Left	20	2	4	2	4
118	70/63/56	20.76	25.72	4.97	Left	21	2	4	9	18
137	70/63/56	20.21	23.77	3.56	Right	21	2	4	5	10
146	70/63/56	20.42	22.37	1.95	Right	21	1	2	–	–
81	70/63/56	25.66	29.14	3.48	Right	26	5	10	23	46
79	70/63/56	25.24	26.93	1.69	Right	26	12	24	–	–
105	59.4/54.12	25.47	27.82	2.35	Left	26	1	2	–	–

The difference ΔD_mean_ is calculated as Final − Initial. The fractions at which DE_0_ and DE_10_ are reached, and their respective cumulative treatment dose are indicated in the rightmost column. Studies 105, 22, 26, 78, 116, 146, and 79 have PGs that did not reach their respective DE_10_ by end of treatment.

### 3.6 Predictive Model

**Figure 5:**
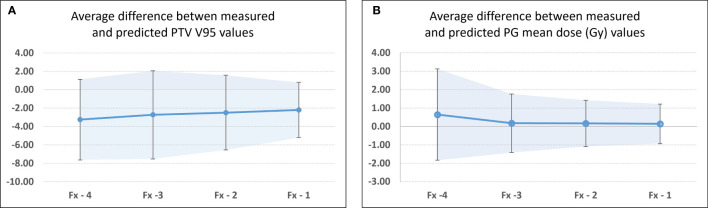
Differences between measured and predicted values at threshold crossing for flagged studies for **(A)** PTV V_95_ and **(B)** PG D_mean_. Fx identified threshold-crossing fraction. Fx-i represents the last fraction that was processed to obtain the model predicted value. In this case, Fx-4 represents the first fraction for which a predicted pDP_sum_ value was available, while Fx-1 identifies the fraction that directly precedes the threshold-crossing fraction. The confidence bands and error bars indicate the 95% confidence interval.

**Figures 4C, D** show the comparison of the model prediction from study 81 (right PG), where the 26 Gy DE_0_ was exceeded at *Fx* = 8 (**Figure 4B**). The predicted trend at fraction 5 (extended dotted line to the right of the white vertical line on **Figure 4C**) indicated that the right PG D_mean_ would cross 26 Gy at fraction *Fx*=8. The accuracy of the prediction model was estimated for all flagged studies except those with identified *Fx* < 5 (in **Tables 4**, **5**) as the model requires a minimum of five treated fractions to generate predictions. The overall difference between measured and predicted PTV V_95_ ranged from −7.6% to 0.0% (mean ± CI_95_, −2.7% ± 4.1%), with the largest differences observed for predictions made four fractions ahead (mean, −3.2%; CI_95_, −7.2%, 1.1%) and the most accurate predictions (mean, −2.2%; CI_95_, −5.2%, 0.8%) obtained closest to the threshold-crossing fraction. **Figure 5A** summarizes the mean V_95_ difference results as a function of temporal proximity defined as the time interval between the threshold-crossing fraction (*Fx*) and the last processed fractions (*Fx − i*) providing the prediction V_95_[*Fx − i*] with *i* progressing from 4 to 1. Uncertainties are reported as 95% confidence intervals (CI_95_). All model predictions overestimated V_95_ coverage values. The variation in prediction accuracy for the PG D_mean_ is presented in **Figure 5B**. The overall difference between the measured and predicted PG D_mean_ values ranged from 0.0 to +5.0 Gy (mean ± CI_95_, 0.2 ± 1.6 Gy). The largest differences were calculated for predictions four fractions ahead (mean, 0.65 Gy; CI_95_, −1.83 Gy, 3.13 Gy), while the highest accuracy was obtained within two (mean, 0.17; CI_95_, −1.09 Gy, 1.43 Gy) to one fraction ahead (mean, 0.14; CI_95_, −0.93 Gy, 1.22 Gy).

### 3.7 Performance of DIR Algorithm

**Figure 6:**
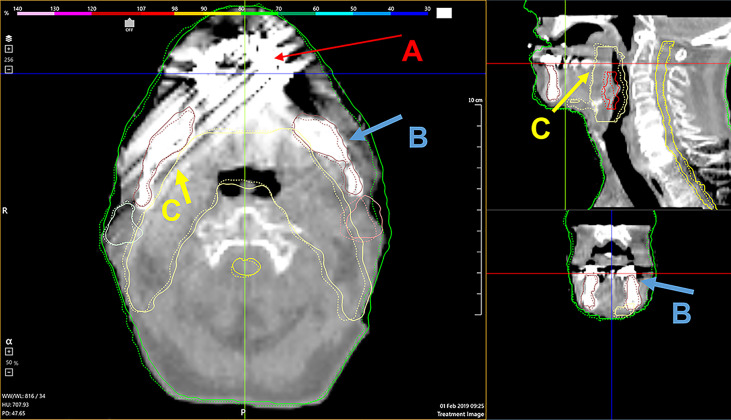
A demonstration of the deformable algorithm’s ability to account for severe CBCT dental implant artifacts [red arrows, **(A)**]. The blue **(B)** and yellow **(C)** arrows indicate the deformed mandible and PTV contour, respectively. Both were minimally affected by the streak artifacts.

The automatically calculated NCC and distance confidence metrics quantitatively identified the structures with questionable deformations and helped to confirm our observations from the qualitative visual review. After several iterations, a single DIR algorithm configuration was found to optimally process all patient data sets without user adjustment. Seventeen patients from our cohort had dental implants that created streak and beam hardening artifacts on daily CBCT images. The registrations seemed robust against imaging artifacts for all 17 patient’s PTV and mandible contours deformations. **Figure 6** illustrates the deformation accuracy of a PTV, mandible, and parotid contours in the presence of severe artifacts. The only issues encountered when reviewing DIR results were the misidentifications of the immobilization mask for the external body contour. These mainly occurred during the second half of treatments, after patients’ weight loss created air gaps between the skin surface and the shell of the mask.

## 4 Discussion

This manuscript is the first to report on an automated platform-agnostic commercial software (RTapp) designed to provide quantitative data to support the adaptive re-planning decision process. The retrospective analysis of 27 HNC patients with the first version of RTapp demonstrated that PTVs and parotid structures would most likely require daily monitoring for adaptation throughout the whole course of RT. The review of daily alignments between CBCTs and the planning CT revealed clear evidence of gradual body weight loss and internal anatomical changes throughout treatment for the 18 flagged patients. Our observations agree with published studies on ART for HNC, which reported on the reduction in target coverage (9, 25, 26) and on the increase in PG dose (18, 36) and spinal cord dose (9, 26) without adaptation. It is highly likely that these 18 patients would have dosimetrically benefited from ART if daily information on the dosimetric impact of anatomical changes was readily available during treatment.

### 4.1 Impact of Sub-Optimal Immobilization

Several patients were flagged for adaptation during the first few treatment fractions. Reviewing daily patient set-up images revealed that their shoulders’ position differed significantly to that seen on their planning CT. The immobilization device clearly failed to provide appropriate and reproducible support for the shoulders, with a direct impact on the dose coverage of the inferior cervical lymph nodes included in IR PTVs and on the PG dosimetry due to the large elasticity of the head and neck tissue, which resulted in a high variability observed in the DP_day_ trends for these patients. In the example from **Figure 4A**, pre-treatment setup images revealed large daily variations in head rotation and shoulder positions, indicating that the patient was able to gradually move within her immobilization mask. This effect of gradual patient weight loss led to the continuous decrease in PTV V_95_ demonstrated by the DP_sum_ trend and crossing the adaptation threshold at fraction 26. Adapting this patient’s RT treatment with a new immobilization would have improved setup reproducibility and raised the PTV coverage.

### 4.2 Predictive Model

The prediction model implemented in RTapp currently relies on a linear regression to forecast the temporal changes of all DP_sum_ values. Our results suggested that such model would most likely overestimate V_95_ values. Clinically, this would result in additional review of target dosimetry in an ART workflow based on RTapp predictions. The accuracy of the model for the PG D_mean_ would currently allow the definition of a 2–3 Gy deviation threshold for plan review based on an initial planned PG D_mean_ in the 20–26 Gy range. Recent decision support methods developed to identify HNC patients for adaptation necessitated large patient cohorts to build prediction models for anatomical changes (37, 38), tumor response (37, 39), and OARs dose accumulation (38, 40). The approach introduced by McCulloch et al. (40) was the closest to that implemented in RTapp. It predicts specific dose metrics values at EOT based on accumulated doses calculated from daily CBCT anatomy. Their model achieved >95% sensitivity and specificity to detect a need for adaptation with predictions based on a minimum of 10 and 15 treated fractions. However, their method involved time-consuming manual steps to generate the predictions and only provided the deviation between planned and received dose at a single time point. In contrast, RTapp relies solely on individual patient data to generate predictions on a per-fraction basis, in real time, without user intervention. The current prediction model accuracy could be improved with the implementation of a multiple regression model accounting for parameters easily available within RTapp—some of which have been shown to correlate with change in OAR dosimetry (41), or with the occurrence of locoregional control for oropharyngeal cancers (36, 42, 43) and incidence of xerostomia (44). Ultimately, machine-learning-based prediction methods might provide the most accurate trend of OAR and targets DPs (45, 46).

### 4.3 Selection of Appropriate Dose Metrics and Deviation Thresholds

The “warning” and “adaptation” threshold for specific DPs are at the core of the automated adaptive decision-making process. There is no consensus on which DPs and deviation thresholds are the most appropriate for triggering adaptation. Therefore, the hypothetical limits to initiate a plan review or adaptive re-planning defined in this work were based on physicians’ constraints provided for treatment planning. McCulloch et al. (40) used a PG D_mean_ of 24 Gy with a 15% (3.6 Gy) threshold based on published NTCP curves (47) and results from a prospective study correlating saliva output with PGs dosimetry (48). Lee et al. (38) analyzed the detection accuracy of their prediction model using the planned PG D_mean_ with a deviation threshold of 10% based on results from Wu et al. (20) but also included 7.5% and 5% under the justification that “physicians would welcome any additional sparing of the PGs.” Brouwer et al. (49) presented a pre-treatment method to select patients for adaptation, based on a PG D_mean_ threshold of 22.2 Gy and a deviation of 3 Gy with a near 80% sensitivity. Other approaches could include the 95% ICRU (3) dose–volume recommendation for minimum PTV coverage or use TCP- and NTCP-derived threshold values for tumors and OARs with specific endpoints. Regardless of the adaptation threshold selection, RTapp supports the monitoring of multiple dose or volume metrics on a per-fraction basis, ultimately allowing the treatment adaptation decision to be quantitatively based on actual patient-specific and daily monitored parameters.

### 4.4 ART Clinical Workflow With RTapp

**Figure 7:**
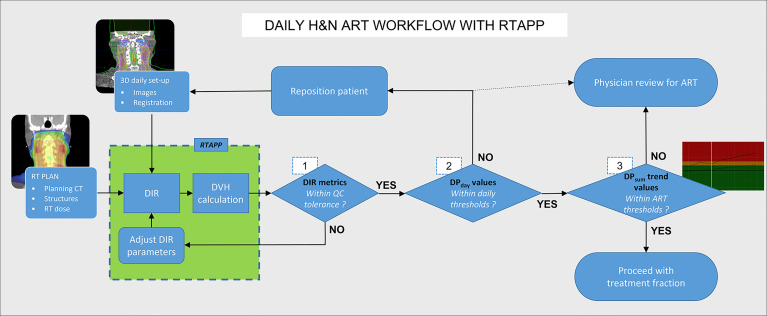
Hybrid ART clinical workflow with RTapp. Decision steps 1–3 occur at the treatment console before treatment delivery. (1) RTapp flags structures with potential deformation issues. (2) The treatment team at the console reviews the DP_day_ values against warning thresholds and might reposition the patient. (3) The current and predicted DP_sum_ trends are reviewed prior treatment to assess whether the treatment adaptation is needed within the next four fractions.

A proposed clinical ART workflow integrating RTapp to monitor patients for potential adaptive re-planning can be divided into three successive steps prior to treatment delivery, as described in **Figure 7**. First, the accuracy of the deformation is evaluated by DIR metrics, with the option to adjust the DIR parameters and reprocess daily setup images instantly. Second, the treating therapy staff at the control console compares DP_day_ values to warning and action thresholds and decide whether to adjust a daily patient setup. Third, the treatment team can be alerted to review trends and predictions of any DP_sum_ if a warning or adaptation threshold is reached. Such information can help with the decision to trigger adaptive re-planning or to carry on with the current treatment plan. Patients’ alignment could potentially be improved daily, or in some cases, urgent adaptive re-planning may take place while minimizing treatment postponement. The proposed workflow is not limited to HNC sites and could be applied to pelvic cancer sites for which differences in daily bladder and rectal filling may impact the dosimetry of targets and OARs, or to non-small cell lung cancers stage II and above, to help with limiting the irradiation of healthy lung tissue caused by shrinking tumors.

### 4.5 Clinical Impact

Online ART is commercially unavailable for gimbal mounted linacs, which constitute the majority of medical linear accelerators installed worldwide (50). In a typical offline ART workflow, the successive steps of processing new 3D patient images (DIR between planning CT and latest CT images), structures re-contouring, re-planning, evaluation of dosimetric and volumetric changes, and QA usually take 2–5 days. RTapp could potentially optimize clinical resources for ART, saving hours of dosimetrist, physicist, and physician work by processing setup images and reporting dosimetric and volumetric changes in less than a minute. Such quantitative information is currently only available on dedicated online ART treatment platforms, which can perform dose recalculation within times ranging from 15 to 60 min (30, 51). The implementation of the predictive model would further optimize the use of resources for adaptive re-planning, by allowing to generate a new treatment plan before a patient meets the requirements to trigger adaptation. In addition, vendor-agnostic ART decision support software applications provide several advantages compared to fully dedicated online ART systems: they are readily deployable with any treatment platform equipped with 3D imaging capabilities and make use of resources already available clinically. Finally, their cost effectiveness is particularly attractive to bring ART to patients from remote rural regions hours away from large academic centers and from low- and middle-income countries.

### 4.6 Limitations

The small sample size resulted in a low number of studies flagged for adaptation and in large CI_95_ for the estimation of the prediction model accuracy. This is in part due to the exclusion of patients with only partial PTV or PG volumes in the CBCT field of view from our original cohort. The limited FOV in the superior–inferior axis from current CBCT imaging systems is an important technological limitation for the proposed method and would require the choice of adaptive DE independent of total structure volumes. However, full structure volumes could be recovered by acquiring two CBCTs, to be merged prior to processing by RTapp at the cost of increased imaging dose, or with new machine learning-based image processing methods to estimate the position of structures outside the FOV of a single CBCT based on the visible anatomy. The first iteration of the RTapp software employed for this study did not have the capability to export the deformed CT and structures data set. Therefore, we could not perform a comparison of RTapp’s estimated dosimetric parameters to those that could have been obtained from an actual dose recalculation with original treatment plan.

### 4.7 Future Work

Once the capability to export deformed data sets is functional, the software performance will be established by evaluating sensitivity, specificity, positive and negative predictive values, and accuracy to determine the need for adaptation for HNC patients based on the DE chosen for this work. Such step is a prerequisite to conduct an observational clinical study aimed at comparing the traditional offline ART workflow, which involves physician-identified cases, to a hybrid RTapp-based workflow such as the one proposed in *Section 4.4*.

## 5 Conclusion

A novel automated decision support software platform for ART was tested retrospectively with 27 HNC patients’ data. Eighteen patients were flagged for adaptation at end of treatment. The trend of PTV coverage and parotid mean doses against specific dose metrics and deviation thresholds on a per-fraction basis demonstrated that RTapp could help identify when to trigger plan adaptation and potentially pro-actively predict when a physician might consider the need for treatment plan adaptation. The tools offered by RTapp have the potential to benefit any clinic equipped with a daily 3D imaging capability without adequate resources to provide ART for their HNC patients. The software platform evaluated provides all the tools and information necessary to design prospective studies aiming to test whether ART will improve outcome both for TCP and NTCP in a diverse range of cancer sites and fractionations.

## Data Availability Statement

The de-identified raw data supporting the conclusions of this article will be made available by the authors, without undue reservation.

## Ethics Statement

The studies involving human participants were reviewed and approved by Loyola University Internal Review Board. Written informed consent for participation was not required for this study in accordance with the national legislation and the institutional requirements.

## Author Contributions

Conception and design of study: SG, AS, AB, and BE. Provision of study material and patients: AB and BE. Data collection: SG and BL. Data analysis and interpretation: SG, AB, BE, BL, and CJ. SG wrote the first draft of the manuscript. All authors contributed to the subsequent manuscript revisions and approved the submitted version.

## Conflict of Interest

Dr. Anand Santhanam is a co-founder of Segana Inc., the company who developed the RTapp software platform evaluated in this work.

The remaining authors declare that the research was conducted in the absence of any commercial or financial relationships that could be construed as a potential conflict of interest.

## Publisher’s Note

All claims expressed in this article are solely those of the authors and do not necessarily represent those of their affiliated organizations, or those of the publisher, the editors and the reviewers. Any product that may be evaluated in this article, or claim that may be made by its manufacturer, is not guaranteed or endorsed by the publisher.
